# A scoping review of the acceptability, feasibility and effectiveness of opt‐out blood‐borne virus (BBV) testing in secondary healthcare settings, in the UK and similar settings, using APEASE criteria

**DOI:** 10.1111/hiv.70284

**Published:** 2026-08-05

**Authors:** Becky Metcalfe, Karen J. Maxwell, Kirsten Trayner, Max Wilkinson, Sharon Hutchinson, Claudia Estcourt, Alison J. Rodger

**Affiliations:** ^1^ School of Health & Life Science, Glasgow Caledonian University Glasgow UK; ^2^ NHS Greater Glasgow & Clyde Glasgow UK; ^3^ Public Health Scotland Edinburgh UK; ^4^ University College London London UK

**Keywords:** BBV, Hepatitis, HIV, opt‐out testing

## Abstract

**Background:**

Earlier diagnosis of blood‐borne viruses (BBVs)—HIV, hepatitis B (HBV) and hepatitis C (HCV)—remains a significant public‐health focus. Opt‐out testing in secondary healthcare settings can improve early diagnosis and linkage to care as part of BBV transmission elimination targets. This scoping review used APEASE criteria (acceptability, practicability, effectiveness, affordability, side‐effects and equity) to evaluate opt‐out BBV testing interventions in emergency departments (EDs) and medical assessment units (MAUs).

**Methods:**

A systematic search of three databases was conducted for peer‐reviewed literature up to 30th June 2025. Inclusion criteria were: (a) opt‐out BBV testing intervention; (b) taking place within a secondary care setting; and (c) in the UK, Ireland, Australia and Canada. Data were extracted and analysed using APEASE criteria.

**Results:**

Twenty‐eight studies met inclusion criteria. Opt‐out testing was acceptable to patients and healthcare professionals, particularly when supported by clear communication, staff training and leadership engagement. Practicability varied by setting; barriers included time constraints, unclear responsibilities and logistical challenges. Automation using electronic systems facilitated implementation. Effectiveness was demonstrated through increased testing uptake and case identification, especially in EDs. Minimal evidence exists on affordability and is likely to vary by population prevalence. No significant side effects (unintended outcomes) were reported, and universal testing was associated with reduced stigma. Opt‐out testing can improve access to testing for underserved populations, supporting its role in promoting health equity.

**Conclusion:**

Opt‐out BBV testing in secondary care settings (EDs and MAUs) is a feasible, effective and equitable intervention when supported by appropriate infrastructure and resources. Efforts should focus on optimizing implementation strategies and extending opt‐out testing, including to lower‐prevalence settings.

## BACKGROUND

Late diagnosis of blood‐borne viruses (BBVs), human immunodeficiency virus (HIV), hepatitis B (HBV) and hepatitis C (HCV) remains a significant public‐health concern. Elimination of BBV transmission is a key global health priority, with the World Health Organization (WHO) and UNAIDS targeting the eradication of HCV [1] and HIV transmission [2] by 2030.

In the UK, HIV and HCV incidence has declined substantially in key populations including gay, bisexual and other men who have sex with men (GBMSM), however marginalized populations, including people who inject drugs, people who are migrants, transgender people and those who experience homelessness, remain at risk of BBV transmission and late presentation highlighting persistent inequalities in access to BBV testing and care [3, 4]. To address these gaps, expansion of routine BBV testing beyond traditional BBV testing healthcare settings (i.e. sexual health or addiction services), has been proposed. One promising strategy is opt‐out testing in emergency departments (EDs) which provides a routine BBV screen to eligible patients (those having venepuncture) unless they decline. In 2022, England introduced opt‐out testing for HIV, HBV and HCV in EDs located in higher HIV prevalence areas. Evaluation after 33 months reported high uptake of testing among those never previously tested, as well as identification of new BBV diagnoses and reengagement of those previously lost to care [5].

Gathering evidence on this approach from the UK and other areas with similar healthcare settings and widening the focus to include evidence from medical assessment units (MAUs) could inform the intervention roll‐out in lower‐prevalence settings, such as Scotland. In Scotland, overall HIV prevalence is low (0.14%), but higher in urban areas of Glasgow and Edinburgh with an estimated 400 people nationally remaining undiagnosed [6]. HBV prevalence is similarly low, with approximately 0.1% of the Scottish population living with chronic HBV (*n* = 5206) [7], compared to prevalence estimate of 0.58% in England [8] where an estimated 55% of those living with chronic HBV are undiagnosed. Scotland has made substantial progress towards HCV elimination [9], yet prevalence among people who inject drugs remains at 15% [10]. New interventions are required if national BBV transmission elimination targets are to be achieved.

In low prevalence areas, BBVs affect key population groups. For example, in Scotland, BBVs disproportionately affect individuals experiencing socioeconomic deprivation, with 54% of those with HBV [7] and 73% of those with HCV [11], residing in Scottish Index of Multiple Deprivation (SIMD) quintiles 1 or 2 (most deprived). Similar disparities are observed in healthcare utilization, with individuals living in the most deprived areas accounting for twice as many ED attendances as those from the least deprived areas and with men more likely than women to present multiple times [12].

There remains limited evidence on where and how best to provide BBV testing interventions in lower BBV prevalence areas and particularly on how to effectively reach currently underserved populations. This scoping review aimed to inform policy recommendations for opt‐out BBV testing in secondary healthcare settings in Scotland and other low prevalence settings, by assessing existing evidence from countries with similar healthcare systems. In the UK (including Scotland), healthcare is predominantly tax‐funded and publicly provided in both community and hospital settings, and similar countries include Ireland, Australia and Canada.

Potential secondary care settings for opt‐out testing include EDs and MAUs. EDs provide emergency and urgent care for all presentations, and patients can self‐present or be referred, and MAUs are hospital‐based units that provide initial care for patients with acute medical conditions—patients may be referred from EDs or other areas (such as primary care). However, only EDs have been included in the successful opt‐out BBV testing programme in England. This review used the APEASE criteria [13] (acceptability, practicability, effectiveness, affordability, side effects and equity), which is a decision‐making framework used to appraise interventions in health policy and implementation science.

## METHODS

### Search strategy and study selection

A systematic search was undertaken of electronic databases: Medline, Embase and Cochrane Library to identify studies evaluating opt‐out BBV testing interventions in secondary health care settings similar to the UK (Supporting Information). The search included articles published between 1 January 2011 and 30 June 2025. Full search strategies are provided in the Supporting Information. Previous reviews and meta‐analyses were also searched to identify eligible studies, as were reference lists of eligible studies.

The search was conducted and reported according to the Preferred Reporting Items for Systematic reviews and Meta‐Analyses extension for Scoping Reviews (PRISMA‐ScR) Statement (Supporting Information).

### Eligibility criteria

Studies were included if they met all the following criteria:

- Conducted in an ED or MAU secondary healthcare setting in the UK, Ireland, Australia, or Canada or were systematic reviews including studies from these countries
- Focused on opt‐out BBV testing
- Study period after 2008
- Published in English

There were no criteria relating to sample size.

Definition of ‘opt‐opt BBV testing’: In order to capture the full range of non‐targeted BBV testing interventions, this was defined by the authors as any BBV testing approach not targeted towards a specific group of people, risk factor or clinical condition. It is recognized that this is beyond true ‘opt‐out’ testing.

Consent for ‘opt‐out’ testing would be considered implied consent; however, in order to assess evidence around consent in non‐targeted BBV testing interventions, studies which described the intervention as ‘universal’ or ‘opt‐out’ were included even when explicit consent was obtained, either for research ethics requirements or as part of clinical service interventions.

### Screening and selection process

Following removal of duplicates, all studies were screened in two stages. Firstly, titles and abstracts of studies were independently reviewed by two reviewers (BM and AR). To assess eligibility in the second stage, full text screening of potentially relevant studies was undertaken by two reviewers (KM and BM) to determine if inclusion criteria were met. Disagreements at both stages were resolved through discussion with two or more co‐authors.

### Data extraction

Data were extracted by two reviewers (BM and KM) with input from a third reviewer (AR). Extraction focused on study characteristics (country, setting, study design and population) and key findings relevant to implementation outcomes.

### Classification of outcome measures

Data were extracted and categorized using the APEASE criteria [12], which evaluates interventions across six categories: (i) *Acceptability*—Is it acceptable to key stakeholders?; (ii) *Practicability*—Can it be implemented as designed within the intended context, material and human resources? This section included feasibility and barriers to implementation; (iii) *Effectiveness*—How effective is the intervention in achieving desired objectives in the target population?; (iv) *Affordability*—How far can it be afforded when delivered at the scale intended?; (v) *Side effects*—How far does it lead to unintended adverse or beneficial outcomes?; (vi) *Equity*—How far does it increase or decrease differences between advantaged and disadvantaged sectors of society?

Findings were synthesized narratively and mapped to these domains.

## RESULTS

### Identification and characteristics of included studies

Figure 1 summarizes the study selection process and Table 1 presents the characteristics of included studies. In total, 28 studies met the inclusion criteria and were included.

**FIGURE 1:**
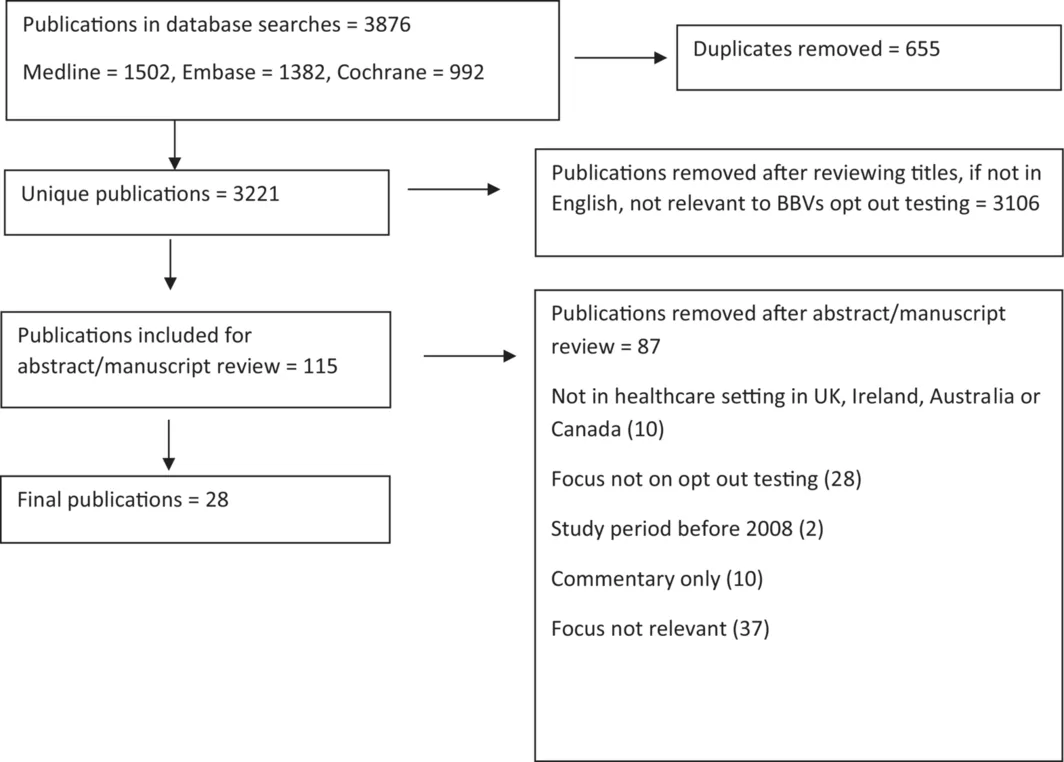
Study selection.

**TABLE 1:** Study characteristics.

Source [ref], year	Publication type, country (area)	Healthcare setting	Study aim	Study intervention	Sample size, duration
Quantitative studies					
Rachman et al [14] (2024)	Single‐site service evaluation quantitative study UK, England (London)	ED	To assess prevalence of HIV infection in ED in an area of high prevalence during opt‐out test pilot.	Opt‐out testing of HIV, HBV and HCV in ED attendees aged over 16 undergoing blood tests in one urban hospital site	BBV tests = 12 208 Individuals tested = 10 647 4 months
Zeng et al. [15] (2023)	Single‐site observational cohort quantitative study UK, England (London)	ED	To identify undiagnosed HCV and HBV. To identify implementation challenges rolling out opt‐out testing in ED.	Opt‐out testing of HBV and HCV in ED attendees undergoing blood tests age over 16 in one hospital site	62% of ED attendees (*n* = 29 279) were eligible 56% of those eligible tested for HBV (*n* = 11 192) or HCV (*n* = 11.215) 4 months
Nebbia et al. [16] (2022)	Single‐site service evaluation quantitative study UK, England (London)	ED	To evaluate opt‐out testing for HBV and HCV in ED.	Opt‐out testing of HBV and HCV in ED attendees aged over 16 undergoing blood tests in one hospital site	46% (*n* = 36 865) of ED attendees (*n* = 81 088) were eligible 75% of those eligible tested for HBV (*n* = 27 646) or HCV (*n* = 27 657) 11 months
Smout et al. [17] (2022)	Single‐site service evaluation quantitative study UK, England (Leeds)	ED	To evaluate the effectiveness and sustainability of opt‐out ED testing for BBVs.	Opt‐out testing of HIV, HBV and HCV in ED attendees aged 16–65 years undergoing blood tests in one urban hospital site	25% (*n* = 28 178) of ED attendees (*n* = 112 479) were eligible of those eligible 60% tested for HCV (*n* = 16 927) and 58% HBV (*n* = 16 407) 9 months
Marchant et al [18] (2022)	Single‐site service evaluation quantitative study UK, England (London)	ED	To evaluate an ED opt‐out testing programme using biochemistry samples for HIV testing.	Routine testing of HIV in ED attendees aged 16–59 undergoing blood tests in one urban hospital site	Of those eligible (*n* = 110 683), 70% were tested (*n* = 78 333) 3 years
Grant et al. [19] (2020)	Single‐site service evaluation quantitative study Ireland (Dublin)	ED	To determine opt‐out BBV testing acceptance, yield and impact on follow‐up care.	Opt‐out testing of HIV, HBV and HCV in adult ED attendees undergoing blood tests in one urban hospital site	63% (*n* = 88 854) of ED attendees (*n* = 140 550) were eligible Of those eligible 62% (*n* = 54 817) were tested 36 months
Gustafson et al. [20] (2020)	Multi‐site service evaluation quantitative study Canada (Vancouver)	ED, MAU, Hospital inpatients	To evaluate the acceptability, feasibility, effectiveness and sustainability of routine HIV testing for inpatient and ED admissions.	Routine HIV testing for inpatient and ED admissions in 3 hospital sites	HIV tests performed =114 803 Of those eligible (*n* = 12 996), 45% (*n* = 5876) were offered an HIV test and 96.9 (*n* = 4935) were tested. 4 years
Allen et al. [21] (2019)	Single‐site cross‐sectional observational quantitative study Ireland (Galway)	MAU	To assess feasibility of opt‐out BBV testing in MAU. To describe prevalence in the study population.	Opt‐out testing of HIV, HBV and HCV in MAU admissions aged over 16 undergoing blood tests in one hospital site	Of those eligible (*n* = 4793) 40% were tested (*n* = 1936) 44 weeks
Bradshaw et al. [22] (2018)	Single‐site quality improvement quantitative study UK, England (London)	ED	To assess the feasibility of opt‐out BBV testing (HIV, HCV and HBV) in the ED To ascertain seroprevalence for these three BBVs in this setting.	Routine testing of HIV in ED attendees aged 16–65 undergoing blood tests in one urban hospital site	Of those eligible (*n* = 25 520) 24% (*n* = 6108) were tested 55 weeks
Hempling et al. [23] (2016)	Single‐site cross‐sectional observational quantitative study UK, England (London)	ED	To determine the feasibility and acceptability of HIV testing in an ED	Opt‐out testing of HIV in ED attendees aged 18–65 undergoing blood tests in one hospital site	35% (*n* = 8404) of ED attendees (*n* = 24 171) eligible 67% of those eligible (*n* = 5657) were included in study 48% (*n* = 2697) offered HIV test and 65% (*n* = 1747) were tested 3 months
O'Connell et al. [24] (2016)	Single‐site cross‐sectional observational quantitative study Ireland (Dublin)	ED	To assess the feasibility and acceptability of universal opt‐out HIV, HBV and HCV testing in the ED. To describe the prevalence of blood‐borne viruses in this population.	Opt‐out testing of HIV, HBV and HCV in ED attendees aged over 18 undergoing blood tests in one urban hospital site	50% (*n* = 19 980) of ED attendees (*n* = 40 000) eligible 50% of those eligible (*n* = 10 000) were included in study 88% (*n* = 8839) individual results analysed 45 weeks
Orkin et al. [25] (2016)	Multi‐site cross‐sectional observational quantitative study UK, England, Scotland	ED	To investigate prevalence of BBVs during a 1‐week routine testing pilot in nine UK EDs.	Opt‐out testing of HIV, HBV and HCV in ED attendees undergoing blood tests in 9 hospital sites	Of those eligible (*n* = 7807) 27% (*n* = 2118) were tested 1 week
Orkin et al. [26] (2015)	Single‐site anonymous seroprevalence study UK, England (London)	ED	To estimate the prevalence of HCV infection in ED in an area of high prevalence.	Not applicable	*n* = 997 samples 2 weeks
Phillips et al. [27] (2014)	Single‐site retrospective cross‐sectional quantitative study UK, England (London)	MAU	To report on the implementation and outcomes of a routine opt‐out HIV testing policy in an MAU.	Routine offer of an HIV test to all 16–79 year olds in one MAU	Of those eligible (*n* = 12 682) 33% (*n* = 4122) were tested for HIV 21 months
Rayment et al. [28] (2013)	Single‐site quality improvement quantitative study UK, England (London)	ED	To develop and deliver a sustainable model of routine HIV testing in ED.	Routine testing of HIV in ED attendees aged 16–65 undergoing blood tests in one urban hospital site	Of those eligible (*n* = 44 582) 10% (*n* = 4327) were tested for HIV 30 months
Chan et al. [29] (2011)	Single‐site observational quantitative study UK, England (London)	MAU	To assess the acceptability of universal opt‐out HIV testing in acute medical admissions.	Routine offer of an HIV test to all 15–59 year olds in one medical admissions unit	Of those eligible (*n* = 101), 83% (*n* = 84) accepted HIV test 2 weeks
Mixed methods and qualitative studies					
Fairhead et al [30] 2025	Multi‐site retrospective evaluation mixed methods study (2) UK, England (London)	ED	To explore opt‐out testing consent models impact on screening in different demographic groups	Opt‐out testing of HIV, HBV and HCV in ED attendees undergoing blood tests in two urban hospital sites	Of those eligible (*n* = 33 388), 59% (*n* = 19 671) tested for at least one BBV *n* = 20 staff interviews 4 months
Blakey et al. [31] (2024)	Multi‐site qualitative study UK, England (London)	ED	To determine attitudes of heath care workers towards BBV screening in EDs post implementation of opt‐out screening.	Opt‐out testing of HIV, HBV and HCV in ED attendees undergoing blood tests in 33 hospital sites	*n* = 610 staff surveyed
Fox et al. [32] (2022)	Single‐site quality improvement mixed methods study UK, England (London)	ED	To describe efforts to increase HIV testing rates in a London ED (inc. opt‐out testing).	Routine testing of HIV in ED attendees aged 16–59 undergoing blood tests in one urban hospital site	Of those eligible (*n* = 46 375) 21% were tested (*n* = 9600) *N* = 40 staff survey respondents 2 years 6 months
Cullen et al. [33] (2019)	Single‐site Qualitative study UK, England (London)	ED	To explore acceptability and feasibility of opt‐out BBV testing in a London ED.	Opt‐out testing of HIV, HBV and HCV in ED attendees aged over 16 undergoing blood tests in one hospital site	*n* = 22 patients and service providers
Crane et al. [34] (2017)	Multi‐site qualitative study UK, England	varied	To examine attitudes of the public and healthcare providers towards the acceptability of specific components of universal BBV testing.	Limited consent computer assisted programme for HIV, HBV, HCV testing	*n* = 119 68 public participants and 51 Healthcare Professionals from range of specialties
Bath et al. [35] (2015)	Single‐site service evaluation mixed methods study UK, England (London)	ED	To review uptake of routine HIV testing in ED	Routine testing of HIV in ED attendees aged over 16 undergoing blood tests in one urban hospital site	Of those eligible (*n* = 9297) 30% (*n* = 2828) were tested *N* = 19 staff completed questionnaire 3 months
Lallenec et al. [36] (2015)	Single‐site observational mixed methods study. Australia (Darwin)	MAU	To determine the uptake, point prevalence and impact on late diagnosis of HIV screening in Acute Medical Admissions.	Routine offer of an HIV test to all adults in one medical admissions unit	Of those eligible (*n* = 10 827) 10% (*n* = 1041) were tested for HIV Tests pre intervention (5.3%, *n* = 287/5429) and post‐intervention (14%, *n* = 754/5398) *n* = 21 medical staff surveyed 16 months
Rayment et al. [37] (2012)	Multi‐site observational mixed methods study UK, England (London)	ED, MAU, Outpatients, Primary care	To assess the feasibility and acceptability, to patients and staff, of routine HIV testing in four non‐specialist settings in areas of high diagnosed HIV prevalence.	Routine offer of an HIV test to all 16–65 year olds in an ED, an acute admissions unit, a dermatology outpatients department and a primary care practice	Of those offered a test (*n* = 6194), 67% (*n* = 4105) accepted HIV test *n* = 144 staff completed questionnaire *n* = 1003 patients completed questionnaire 3 months
Thornton et al. [38] (2012)	Multi‐site qualitative study UK, England (London)	ED, MAU, Outpatients, Primary care	To explore staff attitudes towards implementation of routine HIV testing in four healthcare settings in areas of high diagnosed HIV prevalence.	Routine offer of an HIV test to all 16–65 year olds in an ED, an acute admissions unit, a dermatology outpatients department and a primary care practice	*n* = 85 staff 3 months
Systematic reviews					
Elgalib et al. [39] (2018)	Systematic review UK/USA	ED/MAU	To summarize the published evidence on barriers and facilitators to hospital‐based routine HIV testing in high income countries.	Not applicable	Not applicable
Simmons et al. [40] (2023)	Systematic review Global	ED	To summarize the literature on BBV testing in EDs to evaluate the viability of opt‐out testing in EDs for increasing case finding and linkage to care.	Not applicable	Not applicable
Cost‐effectiveness					
Williams et al. [41] (2022)	Cost‐effectiveness analysis UK	ED	To determine cost‐effectiveness of opt‐out HBV and HCV testing in EDs based on 2 long‐term studies of real world effectiveness of testing in 2 large ED's in the UK.	Not applicable	Not applicable

Abbreviations: BBV, blood‐borne virus; ED, emergency department; HBV, hepatitis B virus; HCV, hepatitis C virus; HIV, human immunodeficiency virus; MAU, medical assessment unit.

Of these, 16 were quantitative studies, of which 12 were conducted in ED settings, three in MAUs and one across ED, MAU and other hospital specialties. Nine studies were qualitative or mixed methods studies, with five focussing on ED, one in MAU and three across a broader range of clinical services. Two systematic reviews [39, 40] included studies from both EDs and MAUs and there was one formal cost‐effectiveness study. See Table 1 for detail.

The majority (*n* = 26) were conducted in or included data from the UK or Ireland reflecting healthcare systems similar to the Scottish context. Five studies specifically explored attitudes towards opt‐out BBV testing, including four focussed on healthcare workers and one in the general public. In terms of BBV focus, 14 studies focussed on HIV only, three assessed interventions for HBV and HCV, one focused solely on HCV and 11 studies evaluated interventions incorporating all three BBVs (Table 1).

Four [14, 15, 30, 31] studies conducted in ED settings evaluated the NHS England national opt‐out testing programme [5], while others described local pilot initiatives implemented before the full roll‐out.

Only one study [21] was conducted in a low‐prevalence setting for BBVs.

### APEASE criteria

A summary of findings from included studies categorized by APEASE criteria is presented in Table 2.

**TABLE 2:** APEASE findings.

Source [ref], year	1. Acceptability (how far is it acceptable to key stakeholders?)	2. Practicability (can it be implemented as designed within the intended context, material and human resources?)	3. Effectiveness (how effective is it in achieving desired objectives in the target population?)	4. Affordability (how far can it be afforded when delivered at the scale intended?)	5. Side effects (how far does it lead to unintended adverse or beneficial outcomes?)	6. Equity (how far does it increase or decrease differences between advantaged and disadvantaged sectors of society?)
Rachman et al [14] (2024)	No data	*Consent*: Notional. Written departmental information. *Education*: No data *Ordering*: No data *Sample*: Additional serum sample *Results*: Managed by specialist team	*Increased testing*: No comparator data *Diagnoses*: 5/88 (6%) of positive HIV tests were new diagnoses *Linkage to care*: 7/8 (88%) who required LTC were successfully linked to care	No data	No data	No data
Zeng et al. [15] (2023)	*Uptake*: 56% eligible patients had BBV test performed	*Consent*: Notional. Written departmental information. *Education*: Propose staff training to increase uptake *Ordering*: No data *Sample*: Additional serum sample *Results*: Managed by specialist team	*Increased testing*: No comparator data *Diagnoses*: HCV Ab– 164 positive; 112 known, 52 new HCV RNA—23 positive; 17 new, 5 known HBsAg—82 positive; 43 known, 39 new *Linkage to care*: HCV 91.3% contacted HBV 97.4% contacted	No data	12% of total samples were repeat o duplicated due to repeat ED attenders	No data
Nebbia et al. [16] (2022)	*Uptake*: 75% eligible patients had BBV test performed	*Consent*: Verbal. Written departmental information. *Education*: Regular ED staff training *Ordering*: Automated test add on, exclusion if tested in previous 6 months *Sample*: Additional serum sample *Results*: Managed by study team	*Increased testing*: No comparator data *Diagnoses*: HCV Ab– 523 (2%) positive; 112 known, 52 new HCV Ag—261 (0.9%) positive; 17 new, 5 known HBsAg—235 (0.9%) positive; 36 known, 39 new *Linkage to care*: HCV 51/99 (52%) who required LTC were successfully linked to care HBV 96/110 (87%) who required LTC were successfully linked to care	Pilot economic evaluation suggested ED HBV/HCV testing is likely to be cost‐effective at a viral prevalence of ≥0.5%	No data	HCV Ag positivity associated with being male, being homeless and living with HIV HBsAg positivity associated with being male, aged 30–49 or 50–69 and non‐white British ethnicity
Smout et al. [17] (2022)	*Uptake*: 60% eligible patients had BBV test performed	*Consent*: Notional. Written departmental information. *Education*: No data *Ordering*: Automated test add on, Optional exclusion if tested in previous 6 months *Sample*: Additional serum sample *Results*: Managed by specialist team	*Increased testing*: No comparator data *Diagnoses*: HIV Ag/Ab– 70 (0.4%) positive; 53 known HCV Ab 342 positive (2.1%) HCV Ag—156 (1%) *Linkage to care*: HIV 16/17 (94%) HCV 58/148 (39%) HBV 27/39 (69%) who required LTC were successfully linked to care	No data	Reduction in stigma	64.7% of HIV diagnoses that required linkage to care were diagnosed with very late‐stage infection and 59% were heterosexual.
Marchant et al [18] (2022)	*Uptake*: 70% eligible patients had BBV test performed	*Consent*: Notional. Written departmental information. *Education*: Close working between specialist and ED teams *Ordering*: Automated test add on *Sample*: Residual biochemistry sample *Results*: Managed by specialist team	*Increased testing*: Testing uptake increased over time *Diagnoses*: HIV Ag/Ab– 1054 (0.4%) positive; 728 (69%) known, 50 (5%) confirmed new *Linkage to care*: No data	No data	259/1054 (25%) of reactive HIV tests were false positives	New HIV diagnoses 80% male 60% at a late stage In addition, only 14% had an AIDS‐defining illness and the diagnosis would have been missed in the remaining 78% in whom HIV was not part of the differential diagnosis or who did not have an indicator condition.
Grant et al. [19] (2020)	*Uptake*: 62% eligible patients had BBV test performed	*Consent*: Verbal. Written departmental information. *Education*: *Ordering*: Automated test add on, exclusion if tested in previous 6 months *Sample*: Additional serum sample *Results*: Managed by specialist team	*Increased testing*: No comparator data *Diagnoses*: Individuals HIV 467 positives, 426 known, 38 new HCV Ab 1744 positives; 1359 known, 182 new HCV PCR 785 positives; 682 known, 103 new HBV sAg– 208 positives; 138 known, 47 new *Linkage to care*: HIV 38/44 (92%) HCV 83% (204/246) HBV 86% (61/71) who required LTC were successfully linked to care	No data	No data	No data
Gustafson et al. [20] (2020)	*Uptake*: 97% of those offered the test accepted *Staff*: 45.2% of eligible patients were offered test	*Consent*: Verbal then notional. Written departmental information. *Education*: Staff education to remove pre‐test counselling perception *Ordering*: Preset order sets *Sample*: Additional serum sample *Results*: Managed by specialist team	*Increased testing*: Increased by over 11 times MAU testing from 3.3% to 22.1% ED from 0.4% to 7.2% *Increased testing* remains high beyond implementation period. *Diagnoses*: HIV 151 new diagnoses *Linkage to care*: 75% (113/151) who required LTC were successfully linked to care	No data	No data	Compared with community those diagnosed in hospital significantly more likely to have acute or late‐stage infection. PWID significantly more likely to be diagnosed in hospital
Allen et al. [21] (2019)	*Uptake*: 40% of eligible were tested *Staff*: Healthcare worker‐associated barriers contributed to uptake being lower than expected	*Consent*: Verbal (research study conditions). Patient information leaflet. *Education*: Propose staff training could improve uptake *Ordering*: No data *Sample*: Additional serum sample *Results*: Managed by study team. Dedicated phone line for results or study queries.	*Increased testing*: No comparative data *Diagnoses*: HIV 1 positive, 1 known HCV Ab 6 positive HCV RNA 3 positive; 3 known HBsAg 4 positives; 3 known *Linkage to care*: 4/4 (100%) who required LTC were successfully linked to care	No data	No data	No data
Bradshaw et al. [22] (2018)	*Uptake*: 24% of eligible were tested *Staff*: Staff rewards resulted in increase in median weekly testing rates	*Consent*: Verbal. Written departmental information. *Education*: Staff training and testing champions. *Ordering*: Automated ‘pop up’ *Sample*: No data *Results*: No data	*Increased testing*: Testing rates improved with time—with weekly performance reviews and financial awards *Diagnoses*: HIV 77 positive; 65 known, 12 new HCV Ab 103 positive; 53 known, 17 new—of which, HCV RNA 6/16 positive HBsAg 32 positives; 14 known, 8 new *Linkage to care*: No data	Calculated cost per new case HIV £2971, HCV £4682, HBV £2683. Cost not including ED or specialist staff time.	No data	No data
Hempling et al. [23] (2016)	*Uptake*: 65% of those offered the test accepted *Reasons for refusal*: Recent test (38%), not wishing to know status (31%) *Staff*: Reasons for not offering test Unable to consent (75%) Forgot to ask (7%) Living with HIV (7%) Other (7%) Previous test (3%)	*Consent*: Verbal. HIV information leaflet. *Education*: Clinical leadership, ED staff training *Ordering*: Automated ‘pop up’ *Sample*: No data *Results*: Managed by specialist team	*Increased testing*: No comparative data *Diagnoses*: HIV positive; 1 new *Linkage to care*: No data	No data	No data	Patients under 47 years more likely to be offered testing. 28–35 year olds most likely, highest at start of pilot Male patients between 28 and 35 yo independently more likely to uptake testing, in multivariate, only being male made you more likely, not age
O'Connell et al. [24] (2016)	*Uptake*: 44% eligible had BBV test *Staff*: Informal ED staff feedback that easier to test as routine compared with having to make a risk‐based judgement	*Consent*: Verbal consent in research study conditions. Written departmental information. *Education*: ED staff training *Ordering*: No data *Sample*: Additional serum sample *Results*: Managed by study team	*Increased testing*: No comparative data *Diagnoses*: HIV 97 positives; 90 known, 7 new *Linkage to care*: 8/8 (100%) who required LTC were successfully linked to care	No data	Results led to implementation of practice as a standard of care. Clinicians reported screening happening at time of presentation allowed immunosuppression or hepatitis to be considered in clinical decision making	Median age 45, 18–102 yo, 50.4% male
Orkin et al. [25] (2016)	*Uptake*: 27% eligible had BBV test	*Consent*: Verbal. Written departmental information. *Education*: ED staff training *Ordering*: No data *Sample*: Additional serum sample *Results*: Managed by specialist team	*Increased testing*: No comparative data *Diagnoses*: HIV 17 positive; 11 known 6 new HCV 39 positive; 24 known 15 new HBV 15 positive; 4 known 11 new *Linkage to care*: Of new diagnosis 21/32 (66%) were successfully linked to care Of known diagnoses, 5/10 (50%) were successfully linked to care	Assuming cost per diagnosis of £7, cost per new case is HIV £2478 HCV £988 HBV £1351	No delay in ED waiting times due to the study.	Higher odds of being tested if male
Orkin et al. [26] (2015)	*Uptake*: Not applicable	*Consent*: Not applicable *Sample*: Anonymized residual biochemistry samples	*Increased testing*: No data *Diagnoses*: HCV Ab seroprevalence 2.65%, sixfold higher than the national prevalence of 0.4%. *Linkage to care*: Not applicable	Propose screening ED attendees aged 25–54 years would be likely to yield the most favourable cost–benefit ratio.	No data	ED attendees 30% Asian and 31% white British but 58% of HCV diagnoses in white British
Phillips et al. [27] (2014)	*Uptake*: 33% eligible patients had HIV test performed	*Consent*: Verbal. Patient information pack *Education*: MAU staff training. Regular specialist team educational visits which are withdrawn gradually *Ordering*: Separate electronic test order prior to venepuncture *Sample*: Additional serum sample *Results*: Managed by specialist team	*Increased testing*: Test rate increased from 33.2% in first 3 months to 41.3% in second 3 months *Diagnoses*: 20 new HIV diagnoses *Linkage to care*: 19/20 (95%) linked to care	No data	Patients diagnosed via opt‐out testing had higher baseline CD4 counts	3 of patients diagnosed were over 59 years old
Rayment et al. [28] (2013)	*Uptake*: 63% of those offered the test accepted *Staff*: Mean proportion offered test was 14% (varying from 6% to 54%)	*Consent*: Verbal. Written departmental information. *Education*: ED staff training. Testing champions, regular teaching and newsletter updates *Ordering*: Electronic prompt *Sample*: Additional serum sample *Results*: Managed by specialist team	*Increased testing*: Testing increased over time *Diagnoses*: HIV 16 new positives *Linkage to care*: 13/16 (81%) linked to care	No data	No data	No data
Chan et al. [29] (2011)	*Uptake*: 83% eligible patients had BBV test performed	*Consent*: Verbal. Written information. *Education*: No data *Ordering*: No data *Sample*: Additional serum sample *Results*: Managed by study team	*Increased testing*: No comparator data *Diagnoses*: No new diagnoses *Linkage to care*: Not applicable	No data	No data	73.1% never tested for HIV before
Mixed methods and qualitative studies						
Fairhead et al [30] 2025	*Staff*: ED staff reported acceptable to patients. Report time as barrier in verbal ‘reminder’ consent model	*Consent*: Verbal ‘reminder’. Written departmental information. Propose implied consent model *Education*: Staff training valued *Ordering*: No data *Sample*: No data *Results*: No data	*Increased testing*: No comparator data *Diagnoses*: HIV—5 new diagnoses HBV—137/19 574 (0.7%) HCV—164/19 623 (0.8%) *Linkage to care*: No data	No data	No data	Reduced testing at peak times (5‐11 pm) Non‐white ethnicity less likely to be tested versus white Men more likely to be tested versus women
Blakey et al. [31] (2024)	*Staff*: 90% agreed worthwhile intervention. 86% reported never or rarely being asked about the intervention by patients. Nurses and other HCWs statistically less likely than doctors to view opt‐out testing as acceptable	*Consent*: 78% HCW agreed to notional. 50.8% suggest verbal reminder at venepuncture. Propose more written departmental information. *Education*: 44% would like more BBV test training, suggesting repeated teaching in concise e‐learning format. 56% ‘very confident’ in discussing BBV testing with patients. *Ordering*: 83% agreed automatic tick boxes for record ‘patient declined’ would be useful *Sample*: Requirement for extra blood tube was a barrier *Results*: Propose specialist team management	*Increased testing*: No data *Diagnoses*: No data *Linkage to care*: No data	Staff concern about repeated testing and potential waste of resources but suggested ways to reduce this, for example, automated blocking of repeat testing.	No data	HCW reported opt‐out benefits of reduced stigma and improving uptake Staff reported benefits of intervention as reducing stigma and HCW anxiety of needlestick incidents
Fox et al. [32] (2022)	*Uptake*: 20% eligible patients tested for HIV *Staff*: Initial ED staff survey revealed 55% (*n* = 22/40) unsure of national HIV testing guidelines	*Consent*: Propose notional. Written departmental information. *Education*: Multidisciplinary departmental ‘champions’ *Ordering*: Preset order sets *Sample*: Additional serum sample *Results*: Managed by specialist team	*Increased testing*: 14% increase in number of patients tested pre and post intervention *Diagnoses*: HIV 74 positive; 63 known, 8 new *Linkage to care*: No data	Cost analysis showed potential annual savings of £70 000–£224 000, not including cost savings in reduced transmission rates.	No data	No data
Cullen et al. [33] (2019)	*Staff and patients*: Majority view BBV testing viewed as acceptable and valuable and practical. Highlight need for change in public norms, perceptions and talk around HIV. Patient receptivity pivots around ‘better to know’	*Consent*: Verbal in simple low fuss terms important. Information—patients hadn't noticed posters. Some concerns about language barriers. Staff reported posters as useful reminder. *Education*: No data *Sample*: Extra vial became habitual for staff. *Results*: ‘No news is good news’ model produced anxiety in patients.	*Increased testing*: No data *Diagnoses*: No data *Linkage to care*: No data	Patients don't wish to waste resources and time—concern around cost‐effectiveness.	No data	ED easier to access that sexual health services for some patients, anxiety of being seen at sexual health clinics, ED supports a protective anonymity. Routine testing could help challenge socially embedded dynamics of affected/unaffected due to risk behaviours
Crane et al. [34] (2017)	*Staff and patients*: Consensus that HIV should be tested like other routine tests. HCPs disagreed that fear of offending patients is a reason not to offer HIV test.	*Consent*: Proposes ‘limited consent’ with written departmental information. HCPs believe patients should be informed verbally at the point of drawing blood. *Education*: No data *Ordering*: No data *Sample*: No data *Results*: No data	*Increased testing*: Public and HCP felt screening programme would detect infections more often. *Diagnoses*: Public agree any loss of patient choice through limited consent was outweighed by benefit of having infections diagnosed earlier. *Linkage to care*: No data	No data	No data	No data
Bath et al. [35] (2015)	*Uptake*: 30% eligible patients had BBV test performed *Staff*: 95% of staff survey respondents agreed routine HIV testing should be permanently rolled out in the ED, 100% if respondents had been trained (uptake rate for survey 9.5%)	*Consent*: Verbal. Written departmental information. *Education*: Collaborative guideline development *Ordering*: No data *Sample*: Additional serum sample *Results*: Managed by specialist team	*Increased testing*: 12 fold increase compared with preintervention *Diagnoses*: HIV; 19 positive, 11 known, 8 new *Linkage to care*: 93% linked to care. 13/14 (93%) who required LTC were successfully linked to care	No data	Staff reported it was easier to do routine testing than on clinical suspicion	No data
Lallenec et al. [36] (2015)	*Uptake*: 14% eligible patients had BBV test performed *Staff*: One third of staff not aware of guideline. One third reported no time for ‘pre‐test counselling’. Half felt HIV testing was indicated One third had concern regarding positive results follow up. No staff reported patient refusal.	*Consent*: Verbal. *Education*: Guideline promoted to medical staff at grand rounds and other routine meetings. Propose sustained promotion and education to maintain testing rate. *Ordering*: Clinician order *Sample*: No data *Results*: Managed by specialist team	*Increased testing*: 9% increase in proportion of medical admissions tested, relative increase 164%. Note then testing decreased again over time. *Diagnoses*: No new diagnoses *Linkage to care*: Not applicable	No data	No data	No data
Rayment et al. [37] (2012)	*Uptake*: 67% of those offered HIV test accepted this *Patients*: 92% agreed test offer was acceptable. Reasons for declining a HIV test (*n* = 265) previously tested recently (54%) not at risk (47%) other health concerns (24%) *Staff*: Supportive of HIV tests in non‐specialist settings	*Consent*: Verbal consent by study staff. Written information. *Education*: Focussed staff training was provided *Ordering*: No data *Sample*: Additional oral fluid or serum sample *Results*: Managed by specialist service	*Increased testing*: No comparator data *Diagnoses*: HIV 8 new positives No positive tests in dermatology outpatients or primary care settings *Linkage to care*: No data	No data	Patients never tested before more likely to accept test	patients from high‐risk demographics had good uptake (MSM, minority ethnic groups) Male and younger patients more likely to accept. Patients approached less likely to be of black African origin than population attending.
Thornton et al. [38] (2012)	*Staff*: Pre‐implementation: Concern that patients may refuse testing due to stigma or lack of knowledge Post‐implementation: Reported it as a useful public‐health intervention. Staff reporting limited patient opposition to testing.	*Consent*: No data *Education*: Identified staff training needs. *Ordering*: No data *Sample*: No data *Results*: Suggest managed by specialist service	*Increased testing*: No data *Diagnoses*: No data *Linkage to care*: No data	Staff requested evidence that routine testing in high‐prevalence areas was a cost‐effective intervention.	Routine offer reduces stigma. Time to discuss and perform test reported as potential barrier in all settings	No data
Systematic reviews						
Elgalib et al. [39] (2018)	*Uptake*: 24% eligible patients had BBV test performed. Patient‐specific factors including female sex, old age and low risk perception correlated with refusal of HIV testing.	*Consent*: Propose verbal consent *Education*: Suggest partnership working with specialist services *Ordering*: No data *Sample*: No data *Results*: Staff fears managing results. Proposed simple results management system.	*Increased testing*: No comparator data *Diagnoses*: No data *Linkage to care*: No data	No data	Report barriers of stigma and privacy to discuss testing	Uptake of HIV testing was higher in younger age groups (4 studies). Male sex was associated with increased uptake of testing (2 studies).
Simmons et al. [40] (2023)	*Staff* report BBV testing in an ED was a feasible and acceptable way of identifying individuals with a BBV.	*Consent*: No data *Education*: No data *Ordering*: No data *Sample*: No data *Results*: No data	*Increased testing*: No comparator data *Diagnoses*: In the 4 UK‐based studies, diagnosed prevalence rates higher in urban ED settings than general UK population (0.4% for HCV, 0.4% for HBV and 0.16% for HIV). *Linkage to care*: 21%–100% linked to care. Staff report intervention can be used for reengagement purposes among those diagnosed but not linked to care	One US study showed cost per QALY close to UK threshold for cost‐effectiveness.	No data	No data
Cost‐effectiveness						
Williams et al. [41] (2022)	*Uptake*: 57% and 75% eligible patients had BBV test performed	*Consent*: No data *Education*: No data *Ordering*: No data *Sample*: Test cost largest cost component across both testing strategies and settings—in large numbers test costs could be negotiated to improve cost‐effectiveness *Results*: Assuming full time band 6 nurse needed to contact patients, budget impact only increased marginally	*Increased testing*: No comparator data *Diagnoses*: Prevalence of infection among ED attendees is key factor in cost‐effectiveness *Linkage to care*: Critical to the cost‐effectiveness of the intervention	HBV and HCV testing in urban EDs is highly cost‐effective in the UK, and can be cost‐effective at relatively low prevalence. Universal testing was highly cost‐effective at a much lower prevalence than the 2% prevalence thresholds currently recommended by ECDC guidelines.	No data	No data

Abbreviations: BBV, blood‐borne virus; ED, emergency department; HBV, hepatitis B virus; HCV, hepatitis C virus; HCW, healthcare workers; HIV, human immunodeficiency virus; MAU, medical assessment unit; MSM, men who have sex with men; PWID, people who inject drugs; AIDS Acquired Immunodeficiency Syndrome; ECDC European Centre for Disease Prevention and Control; HCP, Health Care Practicioner; LTC, linkage to care; PCR, Polymerase Chain Reaction; QALY Quality Adjusted Life Years.

#### Acceptability of an opt‐out BBV testing intervention in ED and MAU

##### Acceptability to those receiving the intervention (patients)

Fourteen quantitative studies measured opt‐out testing acceptability to patients using the proxy measure of testing uptake. Uptake data was available from 19 studies with a broad range of uptake reported, varying by the design of the intervention (Table 2). In ED settings (*n* = 13), testing uptake varied from 20% to 75% and in MAU settings (*n* = 4) three studies reported uptake between 14% and 40% but one study reported 83% [29]. This study [29] included provision of both verbal and written information and BBV specialist staff obtaining verbal consent at the time of taking the test. Another study in both ED and MAU [20] reported HIV testing rates of 22% in MAU and only 7% in ED, but a 97% uptake among those offered the test verbally by a member of the study team. A systematic review [39] reported 24% uptake across the studies reviewed and the cost‐effectivenes analysis [41] reported 57% and 75% in the two sites studies.

In the three qualitative studies [33, 34, 37] examining patient/public acceptability, there was agreement that removing the need to specifically ‘opt in’ to BBV testing was acceptable due to the opportunity for earlier BBV diagnosis. In one study [20] patients viewed testing in EDs/MAUs as a ‘free opportunity’ to undertake BBV testing [33] and in another [37], 92% of those asked found it acceptable. This study [37] reported on reasons for declining a test; of 265 respondents, 54% stated this was due to being tested previously with 47% stating they were not at risk. However, no studies reported on numbers of patients actively declining a BBV test. Only one other study [23] reported on this with 945 participants declining a test—38% citing a recent test and 31% not wishing to know BBV status.

##### Acceptability to those delivering the intervention (healthcare staff )

All studies (*n* = 9) with a qualitative component included staff views of acceptability of the intervention [30, 31, 32, 33, 34, 35, 36, 37, 38] with 8 other studies reporting this in some aspect [17, 21, 22, 23, 24, 28, 33, 39, 40]. Within EDs, opt‐out testing was reported as acceptable to staff, with 95% of staff (doctors and nurses, *n* = 19) in one study agreeing that routine HIV testing should be permanently rolled out [35]. In another study, 86% of ED staff were comfortable offering HIV testing and 81% felt it was appropriate to offer this in the ED [23]. Across EDs and MAUs, staff reported feeling more comfortable with opt‐out testing than targeted approaches, as it reduced stigma [17, 21, 35, 37, 39]. In studies with low testing uptake rates, these were linked to clinicians not offering the test rather than patient refusal [21, 23, 28, 32, 39].

#### Practicability of an opt‐out BBV testing intervention in ED and MAU

Practicability assessed each step of the intervention and therefore this also includes acceptability of how each step in the process was implemented. Overall, most studies suggested that opt‐out BBV testing was achievable across secondary care hospital settings. However, successful implementation required consideration of operational and resource‐related factors, with more studies focussing on this issue in the ED than MAUs (Table 2).

##### Consent

The means of obtaining consent for testing, particularly for HIV, is an important factor in the delivery of the intervention (Table 2). This review defined verbal opt‐out consent to mean when a person is informed that an intervention will proceed unless they object and implied or notional consent to mean consent is assumed without evidence of informed agreement.

Of the studies delivering an intervention (*n* = 20), seventeen studies used explicit verbal opt‐out consent, alongside written information displayed in the department. Three studies used only implied or notional consent based on information displayed in the department [15, 17, 18]. In the studies reporting on testing uptake by those eligible (rather than those offered the test), 56%–70% uptake with implied/notional consent [15, 17, 18] compared to 14%–83% uptake with verbal consent [16, 19, 21, 22, 24, 25, 27, 29, 35, 36].

Four studies specifically explored healthcare workers (HCW) views on BBV opt‐out consent. In one ED study [30], staff described the need to have a verbal discussion as a barrier to testing, especially at busy periods. The largest multi‐site study [31] (*n* = 610 staff) reported that 77.7% of HCPs agreed notional consent was adequate. In another, staff proposed a ‘limited’ model with patients verbally informed at blood draw [34]. Effective communication to patients was important, with seven studies reporting use of departmental posters [15, 20, 24, 25, 27, 33, 35] of which five used multilingual patient leaflets [16, 17, 25, 28, 35, 37]. However, in one study [33], ED patients stated they had not noticed the posters, citing preoccupation with their clinical issue. Overall, patients and staff preferred clear, accessible information [22, 33, 39].

##### Education

A key facilitator increasing staff acceptability is provision of education to improve understanding of the rationale behind BBV opt‐out testing [30, 31, 36]. Education sessions delivered by a BBV specialist team, in collaboration with senior departmental staff, improved staff acceptability [24, 27, 31, 32, 36]. A further facilitator was the level of staff enthusiasm and motivation [27, 39] with local ‘testing champions’ and senior clinical leaders' support seen as key to successful implementation [22, 27, 32, 36, 39]. Barriers related to HIV opt‐out testing included lack of staff awareness of HIV testing guidelines, lack of knowledge about HIV and hesitancy to discuss HIV with patients [18, 28, 32, 36].

##### BBV test ordering and sample

Four studies adapted IT systems to automate test ordering [16, 17, 20, 32], rather than manual ordering, reporting this as key to success [17] with another study using a ‘pop up’ tick box [31]. Three studies noted that the need for an additional or different vial of blood could act as a deterrent [15, 31, 33] and where this was not required [18] staff considered this a facilitator. However, one study reported that over time this extra sample became routine practice [33].

##### Management of BBV test results

Nineteen studies reported management of positive BBV results, which were all implemented via the study team or specialist services rather than the ED or MAU departments. In qualitative studies, ED staff supported this approach for governance and follow‐up of results, with preference for result management outside of the ED [28, 38, 39]. Many studies adopted a ‘no news is good news’ policy, with patients only contacted if results were positive [23, 25, 28, 35, 36]. Close collaboration between EDs, MAUs and sexual health (SH) services was consistently cited as a facilitator [18, 23, 27, 28, 32, 35, 38]. Joint meetings and shared responsibilities, ranging from staff education to results governance and linkage to care, were considered essential for smooth implementation [18, 20, 23, 28, 41]. No studies reported on patient acceptability of methods of results management.

##### Service pressures

Three studies reported that ED staff may perceive opt‐out BBV testing as beyond the scope of emergency care [23, 33, 38] due to the intense clinical pressures [23, 28, 33, 37, 38, 39]. In one study, more than 50% of staff in ED and 40% of MAU staff identified insufficient time as a barrier to opt‐out testing [39] and another demonstrated BBV opt‐out testing in ED was less likely to happen during the busy period between 5 and 9 pm. In MAUs, short patient stays [31], high staff turnover and shift patterns [27] and the involvement of multiple clinical teams [42] were reported as barriers to opt‐out testing intervention success.

#### Effectiveness of opt‐out BBV testing interventions in ED and MAU

The effectiveness of opt‐out BBV testing interventions was reported in 22 studies by rates of testing, seropositivity and linkage to care (Table 2).

##### Increasing testing in EDs

Six studies reported that opt‐out BBV testing significantly increases testing rates in EDs and identified new HIV, HBV and HCV cases [15, 16, 18, 22, 24, 26]. One systematic review reported a 12‐fold increase in testing uptake [35], while another multi‐site study observed an 11‐fold increase [20]. Five studies compared the prevalence of BBVs identified through ED testing with known local population prevalences, showing that BBV prevalence in ED populations was higher than in the general population, particularly in urban areas [15, 24, 25, 26, 35]. A systematic review reported that in 4 UK‐based studies, prevalence rates for HCV, HBV and HIV in ED attendees were higher than the national average [40] and emphasized the importance of considering the demographic profile of the ED attendees, including ethnic diversity, socioeconomic deprivation and rates of injecting drug use [40]. In one study, nearly half of those newly diagnosed with HIV had never previously been tested [18], indicating that ED testing had been successful in reaching individuals who may not perceive themselves to be at risk. There were no studies in EDs in low prevalence settings.

##### Increasing testing in medical assessment units (MAUs)

Four studies reported low uptake of routine BBV screening in MAUs, with correspondingly low case detection rates [21, 29, 36, 42]. Although routine testing increased the number of tests performed, it did not consistently lead to a higher number of new diagnoses. The one study in a low prevalence setting in 2016 [21] reported that the MAU population is older and less representative of the general population, potentially limiting the effectiveness of universal screening. Conversely, one study in a high‐prevalence area in London identified 20 new HIV diagnoses of 4122 tests conducted [27]. A systematic review reported that testing uptake in UK MAUs (70%–84%) (*n* = 3) was higher than in EDs (62%–63%) (*n* = 2) [39] indicating that although case detection may be lower, MAUs can achieve good coverage.

##### Increasing diagnoses of BBVs

Of the studies delivering an intervention, five of the mixed method studies [30, 32, 35, 36, 37] and fifteen of the quantitative studies [14, 15, 16, 17, 18, 19, 20, 21, 22, 23, 24, 25, 27, 28, 29] reported on numbers of positive tests and new BBV diagnoses with only one study [29] reporting no new diagnoses. One study [27] of HIV testing only found those diagnosed through the intervention to have higher baseline CD4 counts when compared to those diagnosed via targeted testing. However, three other studies reported high proportions of those diagnosed to have late‐stage infections [17, 18, 20]. The study in a low prevalence setting reported 4 new BBV diagnoses (of 1936 participants).

##### Linkage to care

Data on successful engagement in care for those who required it is detailed in Table 2. An ED‐based study implementing an active engagement model following a positive HCV test resulted in improved linkage to specialist care [16]. Two studies reported it was more resource intensive to link individuals diagnosed with HCV via the intervention into treatment and care than HIV or HBV, especially among people who inject drugs [16, 17]. Both ED and MAU studies demonstrated that opt‐out testing can also effectively re‐engage patients previously lost to care, although outcomes were dependent on the specific follow‐up model used [14, 15, 19, 24, 27, 28, 37, 39]. In one ED study, 81.5% of patients with known HIV who had disengaged from care were successfully re‐engaged with HIV services through the opt‐out testing intervention [19].

#### Affordability of BBV testing intervention in ED and MAU

Evidence of the cost‐effectiveness of opt‐out BBV testing in secondary health care settings was very limited. Only one formal economic evaluation [41] was identified, with three others reporting cost estimates [16, 26, 32] and only one of these [32] including HIV. Two others reported on ‘cost per case’ for all 3 BBVs [22, 25]. There were no studies reporting on cost‐effectiveness in low prevalence settings.

The UK‐based economic evaluation [41] in 2022, which did not include HIV, found opt‐out ED testing for HBV and HCV to be cost‐effective at prevalence thresholds as low as 0.5% for HCV and 0.25% for HBV, that is, below the 2% prevalence benchmark from the European Centre for Disease Prevention and Control (ECDC) [43], suggesting that opt‐out testing may be economically justified. This analysis emphasized that linkage and reengagement pathways were important determinants of cost‐effectiveness [41].

Four other studies explored perceptions of cost and resource use among patients and healthcare staff views [27, 31, 33, 36]. Some patients and staff expressed concern about ‘wasting resources’ through opt‐out testing [31, 33], while ED staff in a 2012 London‐based study [38] questioned whether routine opt‐out testing was justified without robust cost‐effectiveness evidence. However, this study pre‐dated national ED pilot programmes in England. More recent studies noted that while resource constraints were a concern [33], workflow efficiencies and automation were potential solutions to improve sustainability.

#### Side effects of opt‐out BBV testing interventions in ED and MAU

As in Table 2, 10 studies measured or reported side effects (unintended outcomes) of opt‐out BBV testing. A London‐based study from 2016 [25] reported that introduction of routine opt‐out testing did not increase patient length of stay in the ED, a finding supported by another London study reporting no negative impact on departmental flow or workload [28]. In four studies, both patients and staff generally perceived opt‐out testing as less stigmatizing than targeted approaches [17, 21, 35, 39], noting that it helped normalize BBV testing and integrated it into routine clinical decision making [24]. No study reported on adverse effects on patients after receiving a positive diagnosis of a BBV.

#### Equity impact of opt‐out BBV testing interventions in ED and MAU

A key consideration across studies was the health equity impact of opt‐out BBV testing interventions, specifically whether they reduced or exacerbated disparities between population groups. Three studies suggested that routine opt‐out testing interventions, particularly in EDs, improved access to testing, diagnosis and care among marginalized groups who traditionally face barriers to healthcare engagement [16, 17, 25]. One Canadian study [20] reported that people who inject drugs were more likely to be diagnosed in hospital than community settings. In one UK study from Leeds (2022) [17], 59% of new HIV diagnoses made through ED opt‐out testing were heterosexual individuals with late‐stage infection. Similarly, a multi‐site UK study involving EDs, MAUs and outpatient departments found that individuals who had never previously tested for HIV were more likely to accept testing in non‐specialist settings [37]. In an MAU‐based intervention, it increased the proportion of women and patients of white ethnicity tested for HIV by 41%, suggesting a reduction in demographic disparities in testing uptake. Conversely, one study reported that verbal consent models perpetuate inequities, as they can be influenced by staff‐based risk assessments [30].

## DISCUSSION

Implementing BBV testing in EDs and/or MAUs is a complex intervention that must function in a highly pressurized clinical environment while reaching populations at risk. Although evidence from England has demonstrated the effectiveness of opt‐out testing programmes in EDs located in high and very high HIV prevalence areas [5], important evidence gaps remain regarding the feasibility and value of implementing non‐targeted and opt‐out BBV testing in low prevalence settings. In addition, while EDs have been the primary focus of opt‐out testing initiatives, MAUs may represent another important clinical setting for expanding routine BBV testing. Using the APEASE framework, this scoping review synthesized evidence on non‐targeted and opt‐out BBV testing in EDs and MAUs across the UK and comparable healthcare systems to inform the potential implementation of opt‐out BBV testing programmes in low prevalence areas.

Acceptability was reported as consistently high across studies, with staff and patients generally supportive of opt‐out testing models. Facilitators of acceptability included adequate staff training, visible leadership support and accessible patient information explaining the testing process and consent model. The widespread preference for notional or implied consent, supported by written or digital information in waiting areas and verbal explanation when required, aligned closely with NHS England good practice guidance for BBV opt‐out testing [44]. These findings reinforce that clear communication with patients and staff engagement are critical to mitigating concerns around patient autonomy/consent and staff workload, particularly in busy clinical environments. No studies reported accurately on numbers of patients who declined the intervention, and only two captured reasons for declining from patients; therefore, evidence is limited in this area.

Studies highlighted that practicability and operational feasibility were found to depend on careful planning and integration with existing clinical workflows and digital IT systems. Embedding BBV tests into standard blood order sets and using automated electronic prompts increased testing uptake while reducing staff burden. Sites with strong digital infrastructure reported sustained improvements in testing rates and minimized missed opportunities for diagnosis. Whereas variation in IT systems, staffing pressures and laboratory capacity limited implementation in some settings. More recent evidence, beyond the review's search timeframe, further supports this, demonstrating that digital infrastructure and automation are key to the long‐term sustainability of opt‐out testing programmes [45, 46]. The use of a ‘no news is good news’ communication model for negative results, paired with specialist teams managing positive diagnoses, was valued for both efficiency and patient safety.

In terms of effectiveness, our review found good evidence that opt‐out testing increases BBV testing uptake and case detection compared with targeted or risk‐initiated approaches, though the magnitude of benefit varied with local BBV prevalence. Linkage‐to‐care outcomes were inconsistently reported, highlighting the need for more rigorous and standardized monitoring of this key metric across implementation sites. There is a lack of evidence of the effectiveness of this intervention in low‐prevalence settings. This review found limited studies which compared stage of BBV infection diagnosed via intervention compared with other services. However, the English opt‐out programme found that a higher proportion of people were diagnosed with HIV at a late stage of infection via the programme than via other settings (241: 50.7% vs 832: 36.9%) [5].

Evidence on cost‐effectiveness and whether opt‐out BBV testing represented value for money to the NHS is limited, particularly in lower‐prevalence settings such as Scotland, where yield from testing may be lower but public‐health benefits from identifying undiagnosed infections may still be substantial. This is a key evidence gap. Affordability also examined whether financial and resource requirements of opt‐out BBV testing were manageable for the NHS. It was clear that adequate allocation of resources, particularly staffing, consumables, digital infrastructure and laboratory capacity, was critical for successful implementation in the reviewed studies. However, inconsistent funding could pose barriers to sustainability and scale‐up.

We also reviewed side effects (adverse or beneficial unintended outcomes) associated with opt‐out testing but few studies reported significant side effects, though several studies mentioned that a non‐targeted testing approach reduced stigma [17, 31, 38].

Several studies in the review highlighted that EDs serve as critical access points for individuals who may not engage with other healthcare services, including those from socioeconomically disadvantaged backgrounds. This finding highlights the potential for opt‐out testing in EDs to contribute to health equity by improving access to BBV testing and therefore diagnosis and linkage to care. The equity implications are particularly relevant in the UK context, where emergency care use is strongly linked to deprivation, with individuals living in the most deprived deciles attending EDs at more than twice the rate of those from the least deprived areas [12]. Thus, routine opt‐out BBV testing in EDs may be an effective strategy to reach those disproportionately affected by HIV, HBV and HCV but less likely to engage with preventive or community‐based health services. Embedding opt‐out within routine clinical care also has the potential to normalize BBV screening and reduce stigma, which is a persistent barrier to testing in many communities.

There are limitations of this review which should be acknowledged. As a scoping review, our aim was to summarize available evidence rather than conduct formal quality appraisal or meta‐analysis; therefore, the strength of individual study findings was not weighted. Heterogeneity in study design, implementation models, and outcome measures also limited direct comparison across studies. Additionally, most studies included evidence from high‐prevalence or urban settings in England, which may affect generalizability to lower‐prevalence areas. Finally, eligibility restrictions may have led us to omit eligible non‐English or unpublished studies.

## CONCLUSION

By applying the APEASE framework, we identified that implementation of opt‐out BBV testing in EDs and MAUs should focus on embedding testing into routine clinical pathways in a way that is acceptable, practical and sustainable for both patients and healthcare professionals. Services need to ensure adequate staffing, laboratory capacity and financial investment, alongside strong clinical leadership and robust governance arrangements to support safe delivery, linkage to care for those diagnosed and long‐term sustainability. Digital integration with electronic patient records and automated testing prompts is important to streamline processes for staff and improve uptake.

Although current evidence is limited in lower‐prevalence areas, the findings from this review demonstrate that opt‐out testing in EDs and MAUs has the potential to reduce inequalities and support BBV elimination goals by identifying infections among underserved populations who may not otherwise access testing.

The evidence gaps highlighted, particularly in low prevalence settings, highlight that further studies in these areas, including robust evaluation of pilots, are much needed to inform BBV transmission elimination goals in low prevalence settings.

## AUTHOR CONTRIBUTIONS

BM, AR and KM completed the screening and selection process (as per methods). All other authors contributed to the design of study and to the drafting of the manuscript.

## Supporting information


**Data S1.** Preferred Reporting Items for Systematic reviews and Meta‐Analyses extension for Scoping Reviews (PRISMA‐ScR) checklist.

## Data Availability

Data sharing not applicable to this article as no datasets were generated or analysed during the current study.
